# Quality intrapartum care and associated factors in East Africa: multilevel analysis of recent demographic and health survey

**DOI:** 10.3389/fgwh.2024.1507224

**Published:** 2024-12-17

**Authors:** Alemayehu Kasu Gebrehana, Angwach Abrham Asnake, Beminate Lemma Seifu, Bezawit Melak Fente, Mamaru Melkam, Meklit Melaku Bezie, Zufan Alamrie Asmare, Sintayehu Simie Tsega, Yohannes Mekuria Negussie, Hiwot Altaye Asebe

**Affiliations:** ^1^ Department of Midwifery, College of Health Science, Salale University, Fitche, Ethiopia; ^2^ Department of Epidemiology and Biostatistics, School of Public Health, College of Health Sciences and Medicine, Wolaita Sodo University, Wolaita Sodo, Ethiopia; ^3^ Department of Public Health, College of Medicine and Health Sciences, Samara University, Samara, Ethiopia; ^4^ Department of General Midwifery, School of Midwifery, College of Medicine & Health Sciences, University of Gondar, Gondar, Ethiopia; ^5^ Department of Psychiatry, College of Medicine and Health Science, University of Gondar, Gondar, Ethiopia; ^6^ Department of Public Health Officer, Institute of Public Health, College of Medicine and Health Sciences, University of Gondar, Gondar, Ethiopia; ^7^ Department of Ophthalmology, School of Medicine and Health Science, Debre Tabor University, Debre Tabor, Ethiopia; ^8^ Department of Medical Nursing, School of Nursing, College of Medicine and Health Science, University of Gondar, Gondar, Ethiopia; ^9^ Department of Medicine, Adama General Hospital and Medical College, Adama, Ethiopia

**Keywords:** quality, intrapartum care, associated factors, East Africa, multilevel analysis, demographic and health survey

## Abstract

**Background:**

The time during labor and delivery is crucial for the survival of both women and their infants, as complications that occur during this period can significantly increase the risk of morbidity and mortality. In developing nations, women of reproductive age and their infants are still at risk of morbidity and death from complications associated with pregnancy and childbirth. Morbidity and death from complications of pregnancy and childbirth can be prevented through the utilization of quality care during labor and delivery. However, there is limited evidence on the magnitude and factors associated with quality intrapartum care in East Africa. Therefore, this study assessed the magnitude and associated factors of quality intrapartum care among women in East Africa.

**Methods:**

In this study, we used the most recent Demographic and Health Survey (DHS) dataset from 2015 to 2023, covering 11 East African countries. STATA version 18 software was used for data analysis. Multi-level modeling was applied due to the hierarchical or nested structure of DHS data. Variables with a *p*-value of less than 0.25 in the bivariate multi-level logistic regression model were included in the multivariable multi-level logistic regression analysis. Variables with *p*-values less than 0.05 were considered significant factors associated with receiving quality intrapartum care.

**Results:**

The prevalence of receiving quality intrapartum care in East Africa was 56.38% [95% confidence interval (CI): 56.03, 56.7]. Women with primary education [Adjusted Odds Ratio (AOR) = 1.39, 95% CI: 1.33, 1.46], secondary education (AOR = 1.62, 95% CI: 1.53, 1.62), and higher education (AOR = 1.46, 95% CI: 1.33, 1.60), those in the middle (AOR = 1.28, 95% CI: 1.23, 1.34) and rich (AOR = 1.36, 95% CI: 1.31, 1.43) wealth index categories, women with one (AOR = 1.17, 95% CI: 1.09, 1.25) or 2–4 (AOR = 1.22, 95% CI: 1.16, 1.28) living children, those who perceived the distance from the health facility as not a big problem (AOR = 1.28, 95% CI: 1.24, 1.33), and women living in Rwanda (AOR = 1.30, 95% CI: 1.19, 1.41) had higher odds of receiving quality intrapartum care. Residing in rural areas (AOR = 0.82, 95% CI: 0.78, 0.86), and being from Ethiopia, Kenya, Madagascar, Malawi, Mozambique, Tanzania, Uganda, Zambia, or Zimbabwe, were factors negatively associated with receiving quality intrapartum care.

**Conclusion and recommendations:**

Nearly half of the women in East African countries did not receive quality intrapartum care. Both individual and community-level variables were significantly associated with receiving quality intrapartum care in East Africa. Improving the quality of intrapartum care requires enhancing women's education, addressing socioeconomic challenges, and increasing access to health facilities through targeted interventions.

## Background

Intrapartum care refers to the comprehensive support given to women and their fetuses or newborns throughout the entire process of labor and birth, encompassing the first, second, and third stages of labor, as well as the immediate care provided to both the newborn and the mother following delivery (1). The time during labor and delivery is crucial for the survival of both women and their infants, as complications that occur during this period can significantly increase the risk of morbidity and mortality. The well-being of both the mother and child is influenced by the quality of care the woman receives during labor and childbirth.

In developing nations, women of reproductive age and their infants remain at risk of morbidity and death from complications associated with pregnancy and childbirth (2). According to a World Health Organization (WHO) estimate in 2020, an estimated 287,000 women died from maternal causes worldwide, with a global maternal mortality ratio (MMR) of 223 deaths per 100,000 live births. Sub-Saharan Africa (SSA) had the highest MMR at 545, accounting for 70% of global maternal deaths, with Western, Middle, and Eastern Africa being the regions with the highest maternal mortality rates in the world (3).

According to a WHO report, globally, 47% of all deaths in children under five years of age occurred during the neonatal period (the first 28 days of life). In 2022, approximately 2.3 million newborns died, with SSA accounting for 57% of total under-five deaths and having the highest neonatal mortality rate in the world, at 27 deaths per 1,000 live births (4). This makes the neonatal period one of the most vulnerable stages of life and highlights the need for quality intrapartum and neonatal care.

Despite its importance and the fact that most causes of maternal and neonatal mortality are preventable through quality intrapartum care (including skilled birth attendants at delivery, institutional delivery, and early newborn care), the quality of intrapartum care remains low in SSA (5–9). Studies have shown that factors such as a woman's age, level of education, marital status, wealth index, place of residence, country of residence, media exposure, number of living children, number of Antenatal Care (ANC) visits, and distance from health facilities are associated with the quality of intrapartum care (10–15).

Although studies have been conducted in some East African countries to determine the prevalence of quality intrapartum care, they have been limited to small geographical areas and have not been conclusive. Therefore, this study aims to assess the prevalence of quality intrapartum care and its associated factors in East African countries using nationally representative data from each country. The findings of this study will be valuable for planners, policymakers, non-governmental organizations (NGOs), and researchers in identifying factors related to quality intrapartum care, enabling them to implement suitable interventions and improve the quality of intrapartum care.

## Method

### Study design, setting, data source, population, and sampling procedures

The recent Demographic and Health Survey (DHS) dataset from 2015 to 2023, which includes 11 East African countries (Burundi, Ethiopia, Kenya, Madagascar, Malawi, Mozambique, Rwanda, Tanzania, Uganda, Zambia, and Zimbabwe), was used in this study. DHS is conducted by the United States Agency for International Development (USAID) through its DHS Program. The survey collects and analyzes data on population, health, and nutrition in developing countries and provides data on a variety of topics, including fertility, family planning, maternal and child health, gender, HIV/AIDS, and other health-related issues. After it was requested, the data was downloaded from the DHS website (https://dhsprogram.com/). The source population was all women who gave birth within two years preceding the survey in all 11 East African countries. The study population was all women who gave birth within two years preceding the survey in all 11 East African countries in selected enumeration areas. The datasets from individual countries were appended to determine the magnitude and factors associated with uptake of quality intrapartum care in 11 East African countries. In DHS, a two-stage stratified sampling procedure was used to select the study participants. Our study included a weighted sample of 80,615 women who gave birth within two years preceding the survey.

### Outcome variable

The dependent variable for this study was quality intrapartum care. This variable was generated from a combination of three variables, which include health facility delivery, skilled birth assistant at delivery, and breastfeeding initiation within 1 h (1, 15). Skilled birth attendants are healthcare professionals who attend deliveries, including doctors, nurses, midwives, auxiliary midwives, health officers, and health extension workers. Traditional birth attendants, traditional health volunteers, community health volunteers, neighbors, friends, relatives, and others are not considered skilled birth attendants (15, 16). Women who reported delivering with the assistance of a skilled birth attendant, delivering at a healthcare facility, and having their newborns initiate breastfeeding within one hour of delivery were considered to have received quality intrapartum care (15).

### Explanatory variables

Both individual and community-level variables were included in this study following a review of the literature relevant to our study. Community-level variables include residence, distance from health facility, and country of residence. Individual-level variables include age group, women's level of education, partner's level of education, marital status, wealth index, family size, media exposure, number of living children, number of ANC visits, and mode of delivery.

### Statistical analysis

For data analysis, STATA version 18 software was used. Before analysis, sample weighting was applied. To account for the hierarchical or nested structure of DHS data, multi-level modeling was used. Variables with a *p*-value of less than 0.25 in the bivariate multi-level logistic regression model were included in the multivariable multi-level logistic regression. Variables with *p*-values less than 0.05 were considered significant factors associated with receiving quality intrapartum care.

## Ethical considerations

Permission to access the dataset was obtained from the DHS website (https://dhsprogram.com/). Since this study involved secondary data analysis using publicly available survey data from the DHS program, ethical approval and participant consent were not required. The data files do not contain any personal identifiers, such as individual names or household addresses.

## Results

### Characteristics of the respondents

From the weighted sample of 80,615, about a quarter (25.06%) of the respondents were in the age group of 20–24 years. Almost half (49.77%) of the women had attained primary education. The vast majority, 71,692 (88.93%), of the women were married. More than half (52.10%) of the respondents had no media exposure (had neither a television nor a radio). About 35,087 (43.52%) of the women were in the poor wealth index category. Regarding the mode of delivery, 5,660 (7.04%) of the respondents delivered by cesarean section. More than half, 43,926 (55.56%), of the women had four or more ANC visits during pregnancy. More than three-fourths, 63,241 (78.45%), of the women were rural residents, and about 48,326 (59.95%) reported that the distance to a health facility was not a big problem (Table 1).

**Table 1:** Characteristics of women who gave birth within two years preceding the survey in East Africa (weighted *n* = 80,615).

Variable	Frequency	Percent (%)
Socio-demographic characteristics		
Age (in years)		
15–19	6,198	7.69
20–24	20,206	25.06
25–29	20,041	24.86
30–34	16,025	19.88
35–39	11,145	13.82
40–44	5,323	6.60
45–49	1,677	2.08
Level of education		
No education	17,374	21.55
Primary	40,123	49.77
Secondary	19,973	24.78
Higher	3,145	3.90
Marital status		
Single	5,230	6.49
Married	71,692	88.93
Widowed	1,208	1.50
Divorced	2,485	3.08
Wealth index		
Poor	35,087	43.52
Middle	15,601	19.35
Rich	29,927	37.12
Child, pregnancy and health facility-related variables		
Sex of the child		
Male	40,832	50.65
Female	39,783	49.35
Number of living children		
None	489	0.61
One	19,406	24.07
2–4	41,967	52.06
Five and above	18,753	23.26
Community level variables		
Place of residence		
Urban	17,374	21.55
Rural	63,241	78.45
Distance from health facility		
Big problem	32,289	40.05
Not a big problem	48,326	59.95
Country of residence		
Burundi	8,374	10.39
Ethiopia	7,076	8.78
Kenya	4,883	6.06
Madagascar	8,776	10.89
Malawi	12,946	16.06
Mozambique	5,331	6.61
Rwanda	6,131	7.61
Tanzania	5,685	7.05
Uganda	9,687	12.02
Zambia	7,030	8.72
Zimbabwe	4,696	5.82

### Prevalence of quality intrapartum care uptake

**Figure 1:**
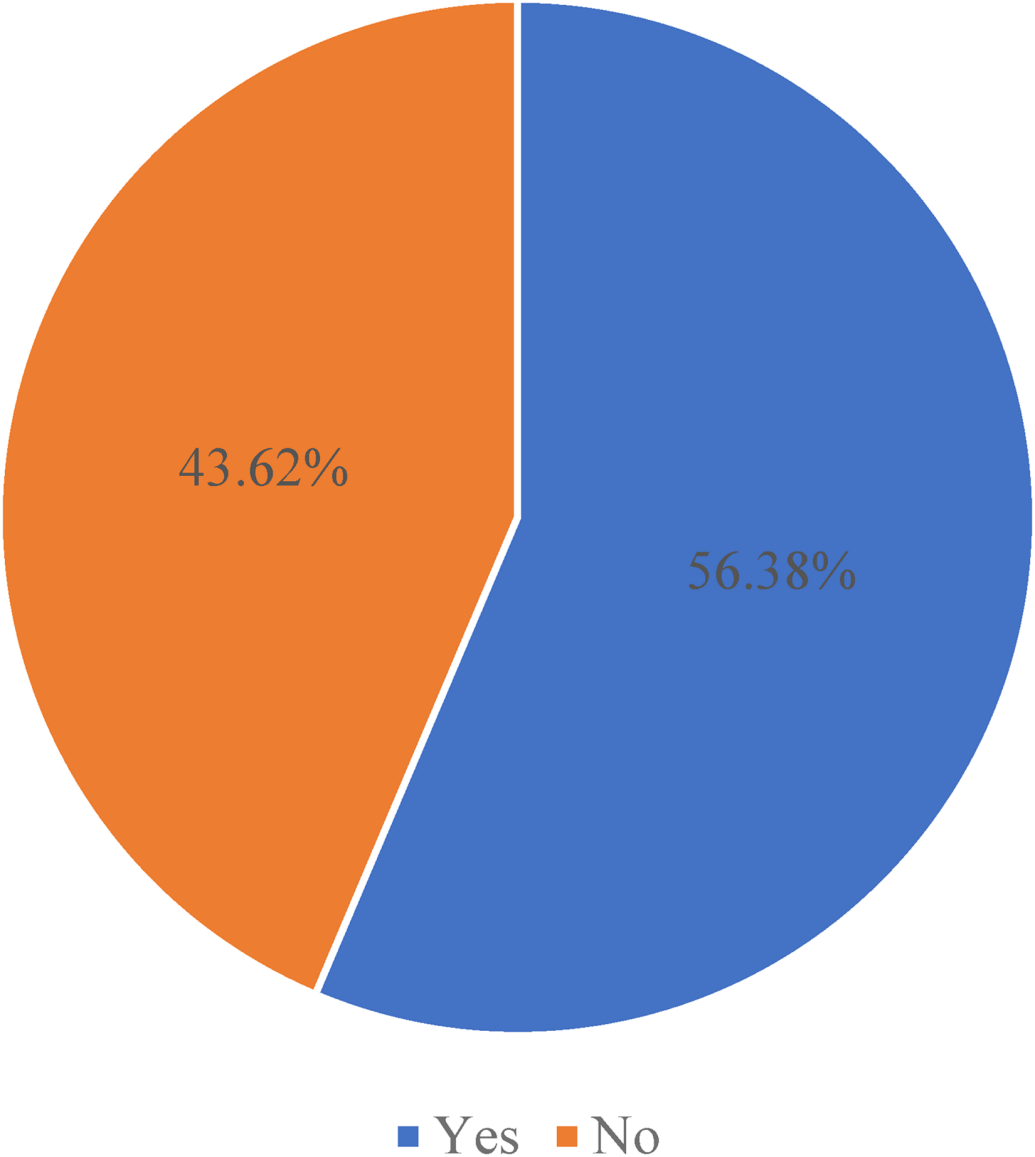
Prevalence of quality intrapartum care uptake in women who gave birth within two years preceding the survey in East Africa.

**Figure 2:**
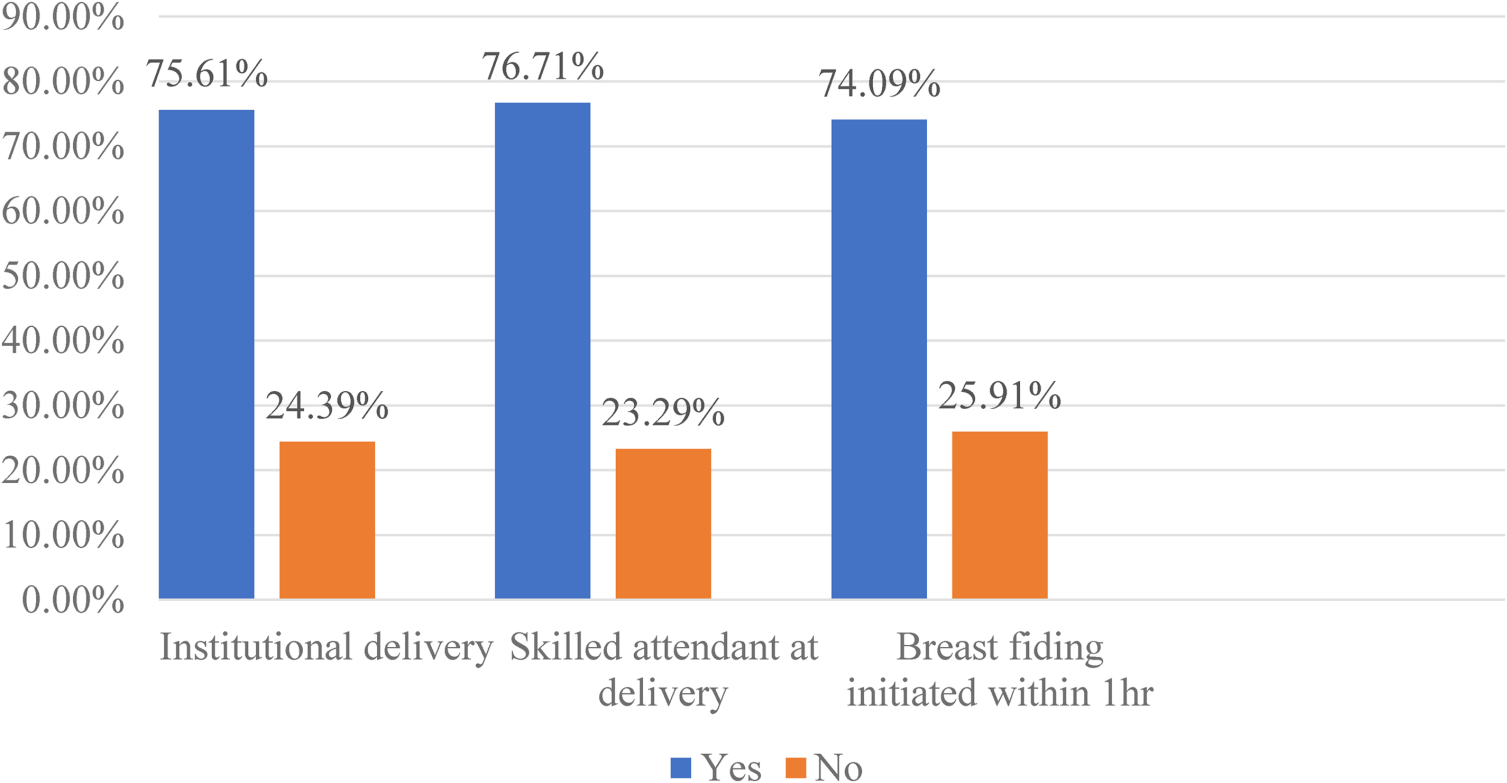
Proportion of uptake of components of quality intrapartum care in women who gave birth within two years preceding the survey in East Africa.

The prevalence of women receiving quality intrapartum care in East Africa was 56.38% (95% CI: 56.03, 56.7) (Figure 1). The highest prevalence of quality intrapartum care was in Malawi (20.51%), while the lowest was in Madagascar (4.92%). About three-fourths (75.61%) of women had institutional deliveries, 76.71% had skilled birth attendants at delivery, and 74.09% initiated breastfeeding within one hour of delivery (Figure 2). More than half (51.98%) of women who attained primary education received quality intrapartum care. About 41.40% of women who received quality intrapartum care were in the rich wealth index category. Nearly one-fourth (24.27%) of respondents who received quality intrapartum care were urban residents.

### Model comparison

For model comparison Deviance, AIC, and BIC were used. Model III, the model with the lowest deviance, AIC, and BIC was considered the best-fit model (Table 2). The null model was fitted without independent variables, while Model I included individual-level variables, Model II included community-level variables, and Model III combined both individual- and community-level variables.

**Table 2:** Model comparison.

	Null model	Model I	Model II	Model III
Log-likelihood	−54,474.04	−52,601.53	−48,209.79	−47,724.34
Deviance	108,948.08	105,203.06	96,419.58	95,448.68
AIC	108,952.1	105,243.1	96,447.58	95,510.68
BIC	108,970.7	105,428.9	96,577.68	95,798.76

### Individual and community-level factors associated with receiving quality of intrapartum care

The results of the multilevel multivariable logistic regression showed that variables such as women's level of education, wealth index, number of living children, type of place of residence, distance from a health facility, and country of residence were significantly associated with the uptake of quality intrapartum care among women in East Africa.

The odds of receiving quality intrapartum care were 1.39 times higher (AOR = 1.39, 95% CI: 1.33, 1.46), 1.62 times higher (AOR = 1.62, 95% CI: 1.53, 1.62), and 1.46 times higher (AOR = 1.46, 95% CI: 1.33, 1.60) among women who attained primary, secondary, and higher education, respectively, compared to those with no formal education. Women in the middle wealth index category had 1.28 times higher odds (AOR = 1.28, 95% CI: 1.23, 1.34) of receiving quality intrapartum care, and women in the rich wealth index category had 1.36 times higher odds (AOR = 1.36, 95% CI: 1.31, 1.43) of receiving quality intrapartum care compared to those in the poor wealth index category. Compared to participants with five or more living children, those with one child and 2–4 children had 1.17 times (AOR = 1.17, 95% CI: 1.09, 1.25) and 1.22 times (AOR = 1.22, 95% CI: 1.16, 1.28) higher odds of receiving quality intrapartum care, respectively. The odds of receiving quality intrapartum care decreased by 18% (AOR = 0.82, 95% CI: 0.78, 0.86) among women residing in rural areas compared to their urban counterparts. Women for whom distance to a health facility was not a big problem had 1.28 times higher odds (AOR = 1.28, 95% CI: 1.24, 1.33) of receiving quality intrapartum care compared to those for whom distance was a big problem. Compared to women living in Burundi, those residing in Rwanda had 1.30 times higher odds (AOR = 1.30, 95% CI: 1.19, 1.41) of receiving quality intrapartum care, while women in all other countries had lower odds of receiving quality intrapartum care (Table 3).

**Table 3:** Individual and community-level factors associated with receiving quality of intrapartum care among women who gave birth within two years preceding the survey in East Africa.

Variable	COR (95% CI)	AOR (95% CI)
Age of the women		
15–19	1	1
20–24	1.19 (1.12, 1.26)**	0.99 (0.93, 1.06)
25–29	1.24 (1.17, 1.31)**	1.02 (0.95, 1.09)
30–34	1.26 (1.18, 1.34)**	1.05 (0.97, 1.13)
35–39	1.19 (1.12, 1.27)**	1.06 (0.98, 1.16)
40–44	1.12 (1.04, 1.20)**	1.04 (0.94, 1.15)
45–49	0.91 (0.81, 1.01)*	0.91 (0.79, 1.04)
Women level of education		
No education	1	1
Primary	1.86 (1.79, 1.93)**	1.39 (1.33, 1.46)**
Secondary	2.09 (2.00, 2.18)**	1.62 (1.53, 1.62)**
Higher	2.07 (1.91, 2.25)**	1.46 (1.33, 1.60)**
Marital status		
Single	1	1
Married	0.89 (0.84, 0.94)**	1.06 (0.99, 1.13)
Widowed	0.84 (0.74, 0.95)**	0.99 (0.86, 1.14)
Divorced	0.96 (0.87, 1.06)	1.03 (0.92, 1.15)
Wealth index		
Poor	1	1
Middle	1.43 (1.38, 1.49)**	1.28 (1.23, 1.34)**
Rich	1.82 (1.76, 1.88)*	1.36 (1.31, 1.43)**
Number of living children		
Five and above	1	1
None	0.95 (0.79, 1.14)	0.91 (0.74, 1.12)
One	1.33 (1.28, 1.39)**	1.17 (1.09, 1.25)**
2–4	1.37 (1.32, 1.42)**	1.22 (1.16, 1.28)**
Place of residence		
Urban	1	1
Rural	0.65 (0.63, 0.68)**	0.82 (0.78, 0.86)**
Distance from health facility		
Big problem	1	1
Not a big problem	1.49 (1.45, 1.54)**	1.28 (1.24, 1.33)**
County of residence		
Burundi	1	1
Ethiopia	0.13 (0.12, 0.14)**	0.14 (0.13, 0.15)**
Kenya	0.32 (0.29, 0.34)**	0.28 (0.26, 0.30)**
Madagascar	0.09 (0.09, 0.11)**	0.09 (0.08, 0.09)**
Malawi	0.99 (0.93, 1.06)	0.92 (0.86, 0.98)**
Mozambique	0.32 (0.30, 0.35)**	0.29 (0.27, 0.32)**
Rwanda	1.54 (1.42, 1.68)**	1.30 (1.19, 1.41)**
Tanzania	0.56 (0.52, 0.61)**	0.49 (0.45, 0.53)**
Uganda	0.34 (0.32, 0.37)**	0.31 (0.29, 0.33)**
Zambia	0.62 (0.58, 0.67)**	0.53 (0.49, 0.58)**
Zimbabwe	0.36 (0.33, 0.39)**	0.26 (0.24, 0.28)**

* *p* < 0.05.

** *p* < 0.01.

## Discussion

The prevalence of receiving quality intrapartum care in East Africa was 56.38% (95% CI: 56.03, 56.7). This finding was higher than those reported in various studies conducted in Ethiopia, including the North Achefer District (27.3%) (10), North West Ethiopia (13%) (11), Tigray (29.2%) (17), and Tanzania (14%) (14). The observed difference in the prevalence of quality intrapartum care may be attributed to the use of nationally representative data in the current study. Previous studies were limited to specific health facilities in smaller geographical areas. This broader scope in the current study likely provides a more comprehensive view of quality care across the region. The discrepancy may also stem from differences in how quality was assessed. This study focused on three key variables from the DHS, while previous studies used checklists that considered additional factors, such as intrapartum care interventions during labor and delivery, starting from the time of admission, as well as the availability of health services.

The odds of receiving quality intrapartum care were higher in women who attained primary, secondary, and higher education levels compared to those with no formal education. This finding was consistent with the studies conducted in Ethiopia (15) and Uganda (13). A possible reason for this could be that women with higher levels of education are more likely to utilize maternal health services, such as ANC, skilled birth attendance, and postnatal care, due to greater awareness of the importance of timely and appropriate care (18, 19). This increased knowledge leads to a higher likelihood of receiving quality intrapartum care compared to women with no formal education. Women in the middle and rich wealth index categories had higher odds of receiving quality intrapartum care compared to those who were in the poor wealth index category. This finding was supported by studies conducted in Ethiopia (15), Gabon (20), Angola (21), another study in SSA (22), and a study in low- and middle-income countries (23). This might be because wealthier women receive higher quality intrapartum care due to better access to healthcare, the ability to afford skilled services, greater awareness of maternal care, and stronger social support networks (24, 25). Compared to women with five or more living children, those with one and 2–4 living children had higher odds of receiving quality intrapartum care. This finding aligns with studies conducted in Ethiopia (15), low- and middle-income countries (23), Cameron (26), and China (27). One possible reason is that families with many children, especially those in the lower income quintile, face greater financial challenges, making it harder for mothers to afford healthcare costs, resulting in difficulties in accessing quality intrapartum care (23, 27). However, this finding contradicts results from the study in SSA, which revealed that the number of children was positively associated with access to maternal healthcare service (28). This could be because, as women become mothers, they gain a greater understanding of the importance of quality maternal care, which often drives them to seek better healthcare options, recognizing that proper support and resources are essential for their health and the health of their children during this critical time (28, 29).

The odds of receiving quality intrapartum care were lower in women residing in rural areas compared to their urban counterparts. This finding was supported by studies conducted in Hadiya Zone, Ethiopia (30), Low- and Middle-Income Countries (23), Southern Ethiopia (31), and India (32). This could be because urban women are more likely to seek healthcare services than their rural counterparts due to higher levels of knowledge and awareness, better access to government and private healthcare facilities, greater emphasis on education, and the advantages of shorter distances, improved infrastructure, and public transportation (29, 32, 33). The odds of receiving quality intrapartum care were higher in women for whom distance from a health facility was not a big problem, compared to those for whom distance from a health facility was a big problem. This finding was congruent with studies in Ethiopia (5, 10, 34, 35), Nepal (36), and Zambia (37). This could be due to longer distances deterring women from seeking timely medical assistance during labor and delivery, leading to potential delays caused by increased travel time, transportation costs, and the physical challenges associated with reaching healthcare facilities.

Women who live in Rwanda had higher odds of receiving quality intrapartum care, while those who live in Ethiopia, Kenya, Madagascar, Malawi, Mozambique, Tanzania, Uganda, Zambia, and Zimbabwe had lower odds of receiving quality intrapartum care compared to women who live in Burundi. This might be due to Rwanda's strong health system, community-based healthcare initiatives, and significant progress in reducing maternal mortality while improving access to skilled birth attendants (38).

## Strengths and limitations

Nationally representative data from 11 East African countries were used in the study. Both individual and community-level variables were considered. Multilevel modeling was employed to account for the hierarchical nature of the data. Despite these strengths, the study has some limitations. The cross-sectional nature of the data prevents the identification of cause-and-effect relationships. Additionally, since the DHS dataset lacks certain variables, such as interventions during labor and delivery that were not included in the analysis, the magnitude of quality intrapartum care may be overestimated.

## Conclusion and recommendations

Our study results revealed that nearly half of the women in East Africa did not receive quality intrapartum care. Women's education level, wealth index, and number of living children were individual-level factors significantly associated with the uptake of quality intrapartum care. Residence, perceived distance from health facilities, and country of residence were community-level variables significantly associated with the uptake of quality intrapartum care. Improving the quality of intrapartum care requires enhancing women's education, addressing socioeconomic challenges, and increasing access to health facilities through targeted interventions.

## Data Availability

Publicly available datasets were analyzed in this study. The datasets analyzed during this study are publicly available from the DHS website (https://dhsprogram.com/).

## References

[1] World Health Organization. WHO recommendations: intrapartum care for a positive childbirth experience 2018. Available online at: https://www.who.int/publications/i/item/9789241550215 (accessed September 08, 2024). 30070803

[2] Kassebaum NJ Bertozzi-Villa A Coggeshall MS Shackelford KA Steiner C Heuton KR Global, regional, and national levels and causes of maternal mortality during 1990–2013: a systematic analysis for the global burden of disease study 2013. Lancet. (2014) 384(9947):980–1004. 10.1016/S0140-6736(14)60696-6 24797575 PMC4255481

[3] World Health Organization. Trends in maternal mortality 2000 to 2020: estimates by WHO, UNICEF, UNFPA, World Bank Group and UNDESA/Population Division 2023. Available online at: https://www.who.int/publications/i/item/9789240068759 (accessed September 10, 2024).

[4] World Health Organization. Newborn mortality 2024. Available online at: https://www.who.int/news-room/fact-sheets/detail/newborn-mortality#:∼:text=Overview,in%20child%20survival%20since%201990 (accessed September 10, 2024).

[5] Fisseha G Berhane Y Worku A Terefe W. Distance from health facility and mothers’ perception of quality related to skilled delivery service utilization in northern Ethiopia. Int J Womens Health. (2017) 9:749–56. 10.2147/IJWH.S140366 29042819 PMC5633329

[6] van den Broek NR Graham WJ. Quality of care for maternal and newborn health: the neglected agenda. Bjog. (2009) 116(Suppl 1):18–21. 10.1111/j.1471-0528.2009.02333.x 19740165

[7] Abebaw WA Wolde HF Tilahun WM Gebreegziabher ZA Teshome DF. Quality of childbirth care and its determinants along the continuum of care among pregnant women who gave birth vaginally in Gondar town public health facility, northwest Ethiopia, 2022: generalised structural equation modelling. BMJ Open. (2024) 14(4):e073199. 10.1136/bmjopen-2023-073199 38580371 PMC11002431

[8] Abebe AH Mmusi-Phetoe R. Quality of obstetric and newborn care in health centers of Addis Ababa city: using the WHO quality framework. BMC Health Serv Res. (2023) 23(1):459. 10.1186/s12913-023-09414-7 37161461 PMC10169211

[9] Housseine N Punt MC Mohamed AG Said SM Maaløe N Zuithoff NPA Quality of intrapartum care: direct observations in a low-resource tertiary hospital. Reprod Health. (2020) 17(1):36. 10.1186/s12978-020-0849-8 32171296 PMC7071714

[10] Asmare Y Tilahun T Debela Y Eshetie Y Minuye B Yalew ZM Quality of intrapartum care at public health institutions of north Achefer district, north west Ethiopia: a mixed method study. BMC Pregnancy Childbirth. (2022) 22(1):626. 10.1186/s12884-022-04907-5 35941583 PMC9358859

[11] Asrese K. Quality of intrapartum care at health centers in Jabi Tehinan district, North West Ethiopia: clients’ perspective. BMC Health Serv Res. (2020) 20(1):439. 10.1186/s12913-020-05321-3 32429907 PMC7236140

[12] Berhane B Gebrehiwot H Weldemariam S Fisseha B Kahsay S Gebremariam A. Quality of basic emergency obstetric and newborn care (BEmONC) services from patients’ perspective in Adigrat town, Eastern Zone of Tigray, Ethiopia. 2017: a cross sectional study. BMC Pregnancy Childbirth. (2019) 19(1):190. 10.1186/s12884-019-2307-6 31146729 PMC6543605

[13] Kigenyi O Tefera GB Nabiwemba E Orach CG. Quality of intrapartum care at Mulago national referral hospital, Uganda: clients’ perspective. BMC Pregnancy Childbirth. (2013) 13(1):162. 10.1186/1471-2393-13-162 23941203 PMC3751160

[14] Larson E Hermosilla S Kimweri A Mbaruku GM Kruk ME. Determinants of perceived quality of obstetric care in rural Tanzania: a cross-sectional study. BMC Health Serv Res. (2014) 14(1):483. 10.1186/1472-6963-14-483 25326007 PMC4283093

[15] Negash WD Asmamaw DB Wassie GT Azene AG Eshetu HB Terefe B Less than one in four mothers get quality intrapartum health care services in Ethiopia. Sci Rep. (2024) 14(1):4194. 10.1038/s41598-024-54506-x 38378838 PMC10879093

[16] Tessema ZT Tesema GA. Pooled prevalence and determinants of skilled birth attendant delivery in east Africa countries: a multilevel analysis of demographic and health surveys. Ital J Pediatr. (2020) 46(1):177. 10.1186/s13052-020-00943-z 33256803 PMC7708172

[17] Fisseha G Berhane Y Worku A. Quality of intrapartum and newborn care in Tigray, northern Ethiopia. BMC Pregnancy Childbirth. (2019) 19(1):37. 10.1186/s12884-019-2184-z 30658706 PMC6339373

[18] Yadav AK Sahni B Kumar D Bala K Kalotra A. Effect of women’s and partners’ education on maternal health-care services utilization in five empowered action group states of India: an analysis of 13,443 women of reproductive age. Int J Appl Basic Med Res. (2021) 11(4):231–7. 10.4103/ijabmr.ijabmr_121_21 34912686 PMC8633696

[19] Amwonya D Kigosa N Kizza J. Female education and maternal health care utilization: evidence from Uganda. Reprod Health. (2022) 19(1):142. 10.1186/s12978-022-01432-8 35725605 PMC9208099

[20] Sanogo N Yaya S. Wealth status, health insurance, and maternal health care utilization in Africa: evidence from Gabon. BioMed Res Int. (2020) 2020(1):4036830. 10.1155/2020/4036830 32461984 PMC7212326

[21] Aoki A Mochida K Kuramata M Sadamori T Sapalalo P Tchicondingosse L Association between the quality of care and continuous maternal and child health service utilisation in Angola: longitudinal data analysis. J Glob Health. (2023) 13:04073. 10.7189/jogh.13.04073 37565413 PMC10416139

[22] Asefa A Gebremedhin S Marthias T Nababan H Christou A Semaan A Wealth-based inequality in the continuum of maternal health service utilisation in 16 Sub-Saharan African countries. Int J Equity Health. (2023) 22(1):203. 10.1186/s12939-023-02015-0 37784140 PMC10544383

[23] Shanto HH Al-Zubayer MA Ahammed B Sarder MA Keramat SA Hashmi R Maternal healthcare services utilisation and its associated risk factors: a pooled study of 37 low- and middle-income countries. Int J Public Health. (2023) 68:7–8. 10.3389/ijph.2023.1606288 PMC10625904 37936874

[24] Kruk ME Gage AD Arsenault C Jordan K Leslie HH Roder-DeWan S High-quality health systems in the sustainable development goals era: time for a revolution. Lancet Glob Health. (2018) 6(11):e1196–e252. 10.1016/S2214-109X(18)30386-3 30196093 PMC7734391

[25] Ronsmans C Graham WJ. Maternal mortality: who, when, where, and why. Lancet. (2006) 368(9542):1189–200. 10.1016/S0140-6736(06)69380-X 17011946

[26] Mbuagbaw LCE Gofin R. A new measurement for optimal antenatal care: determinants and outcomes in Cameroon. Matern Child Health J. (2011) 15(8):1427–34. 10.1007/s10995-010-0707-3 21057862

[27] Wu X Li L. Family size and maternal health: evidence from the one-child policy in China. J Popul Econ. (2012) 25(4):1341–64. 10.1007/s00148-011-0361-0

[28] Iacoella F Tirivayi N. Determinants of maternal healthcare utilization among married adolescents: evidence from 13 Sub-Saharan African countries. Public Health. (2019) 177:1–9. 10.1016/j.puhe.2019.07.002 31470265

[29] Istifa MN Efendi F Wahyuni ED Ramadhan K Adnani QES Wang J-Y. Analysis of antenatal care, intranatal care and postnatal care utilization: findings from the 2017 Indonesian demographic and health survey. PLoS One. (2021) 16(10):e0258340. 10.1371/journal.pone.0258340 34637462 PMC8509866

[30] Ahmed R Sultan M Abose S Assefa B Nuramo A Alemu A Levels and associated factors of the maternal healthcare continuum in Hadiya zone, southern Ethiopia: a multilevel analysis. PLoS One. (2022) 17(10):e0275752. 10.1371/journal.pone.0275752 36215257 PMC9550044

[31] Yoseph A Teklesilasie W Guillen-Grima F Astatkie A. Individual- and community-level determinants of maternal health service utilization in southern Ethiopia: a multilevel analysis. Women’s Health. (2023) 19:17455057231218195. 10.1177/17455057231218195 PMC10748615 38126304

[32] Paul P Chouhan P. Socio-demographic factors influencing utilization of maternal health care services in India. Clin Epidemiol Glob Health. (2020) 8(3):666–70. 10.1016/j.cegh.2019.12.023

[33] Singh L Rai RK Singh PK. Assessing the utilization of maternal and child health care among married adolescent women: evidence from India. J Biosoc Sci. (2012) 44(1):1–26. 10.1017/S0021932011000472 21933465

[34] Ayehubizu LM Yohannes SH Yadeta ZS Fetene MT. Completion of maternal and child health continuum of care and associated factors among women in Gode district, Shebele Zone, Eastern Ethiopia, 2022. BMC Pregnancy Childbirth. (2024) 24(1):441. 10.1186/s12884-024-06639-0 38914927 PMC11194907

[35] Kebede A Hassen K Nigussie Teklehaymanot A. Factors associated with institutional delivery service utilization in Ethiopia. Int J Women’s Health. (2016) 8:463–75. 10.2147/IJWH.S109498 27672342 PMC5026219

[36] Wagle RR Sabroe S Nielsen BB. Socioeconomic and physical distance to the maternity hospital as predictors for place of delivery: an observation study from Nepal. BMC Pregnancy Childbirth. (2004) 4:1–10. 10.1186/1471-2393-4-8 15154970 PMC425583

[37] Gabrysch S Cousens S Cox J Campbell OM. The influence of distance and level of care on delivery place in rural Zambia: a study of linked national data in a geographic information system. PLoS Med. (2011) 8(1):e1000394. 10.1371/journal.pmed.1000394 21283606 PMC3026699

[38] United Nations International Children’s Emergency Fund. Maternal health-Rwanda (2024). Available online at: https://www.unicef.org/rwanda/topics/maternal-health?items_per_page=10 (accessed October 01, 2024).

